# Compound and acutely ruptured false aneurysm of the brachial artery: a case report

**DOI:** 10.4076/1752-1947-3-6627

**Published:** 2009-06-05

**Authors:** Elias Panagiotopoulos, Efstratios Athanaselis, Charalampos Matzaroglou, Georgios Kasimatis, John Gliatis, Ioannis Tsolakis

**Affiliations:** 1 Department of Orthopaedic Surgery, University Hospital of Patras, T.K. 26500, Greece; 2 Vascular Unit, University Hospital of Patras, T.K. 26500, Greece

## Abstract

**Introduction:**

A patient with a neglected, compound acutely ruptured false aneurysm of the brachial artery which developed after a periprosthetic fracture of the right humerus, is reported.

**Case presentation:**

An 84-year-old Greek woman underwent right shoulder hemiarthroplasty 2 years before a periprosthetic fracture which was treated conservatively in another hospital. After removing the U-slab herself, she noticed the development of an ulcer on the mid-humerus, with continuous oozing but no fever. This led to above-elbow amputation in an attempt to save the patient's life.

**Conclusion:**

It is hoped that by awareness of such a possibility coupled with an early diagnosis based on the clinical picture and imaging modalities, such unfortunate results can be avoided in the future. In case of increasing displacement at the fracture site and excessive local swelling, the possibility of the presence of a false brachial aneurysm should be ruled out despite the presence of normal perfusion of the hand and palpable radial and ulnar pulses.

## Introduction

False aneurysms of peripheral arteries are very rare and in most cases, these are the result of penetrating injuries, such as gunshot or stab wounds, and iatrogenic arterial injury. Fractures as well as blunt trauma have also been reported as causes. These aneurysms are much less frequent in the upper extremity than in the lower extremity [1,2] and they can even cause the loss of the extremity [2]-[6].

A patient with a traumatic false aneurysm of the brachial artery after a periprosthetic fracture of the humerus is presented. Inadequate and insufficient conservative treatment of the periprosthetic fracture led to above-elbow amputation.

## Case presentation

An 84-year-old Greek woman was admitted to our clinic via the accident and emergency department complaining of an extremely swollen and ulcerated mid-humerus after a periprosthetic fracture of her right humerus. Two years previously, she had undergone a hemiarthroplasty for a four-part humeral head fracture in another hospital. According to the patient, the postoperative rehabilitation was fair with painful and restricted motion of the shoulder. Two months before her admittance to the clinic, she sustained a closed, oblique periprosthetic fracture of the humerus after a fall onto the hyper-extended right hand. Initially, the fracture was treated conservatively with a U-slab in the hospital where she underwent the previous shoulder surgery. The patient removed the splint after 10 days without medical consultation. Three weeks before admission to our clinic, she noticed the development of an ulcer on the mid-humerus, with continuous oozing but no fever.

**Figure 1:**
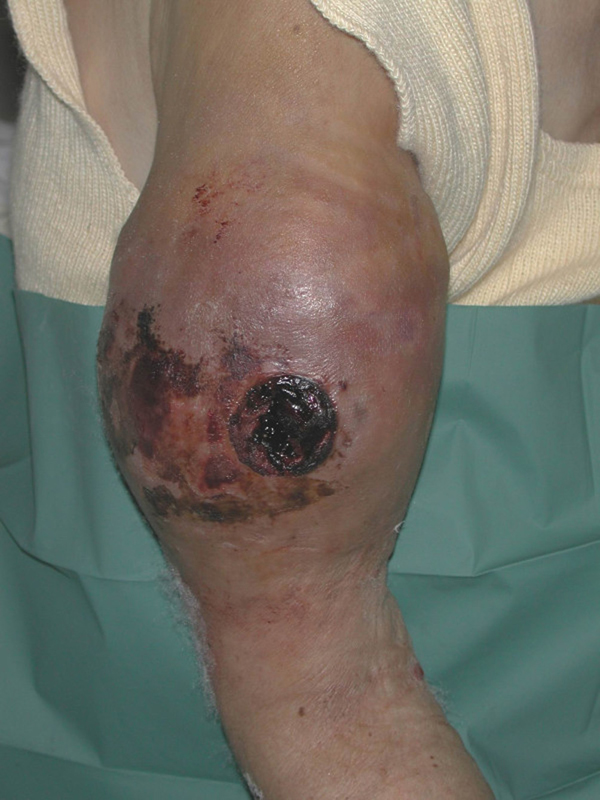
**Photograph showing excessive swelling of the mid-arm as well as ulceration**.

**Figure 2:**
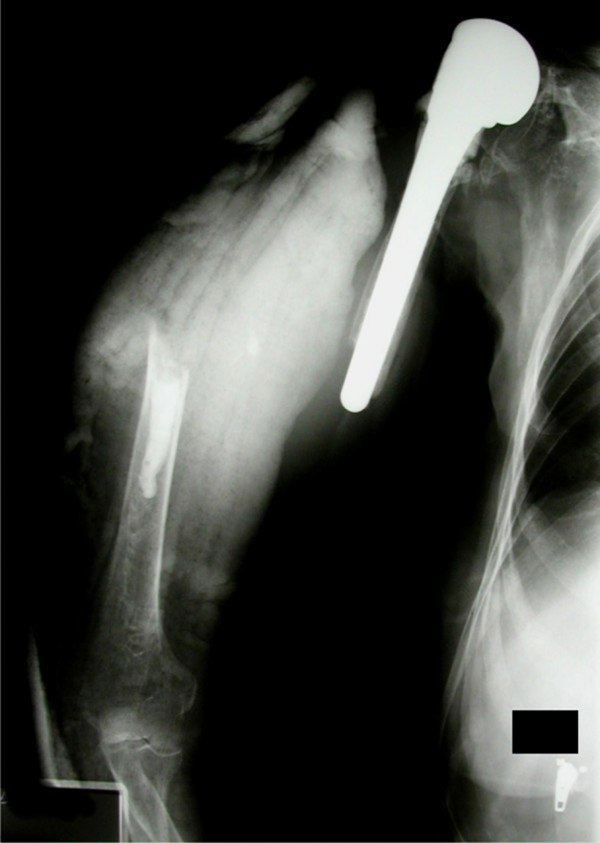
**Plain radiograph showing gross displacement at the fracture site**.

On admission, clinical examination revealed a very painful and ulcerated swelling on her right arm (Figure 1). The ipsilateral forearm and hand were well perfused with normal brachial pulses at the elbow fossa and radial and ulnar pulses on the wrist. There was no neurological deficit but elbow motion was restricted because of the pain. Radiological examination revealed a periprosthetic fracture of the right humerus with approximately 10cm displacement (Figure 2). Laboratory tests revealed severe anemia with hematocrit (Hct) 13.5% and hemoglobin (Hb) 4.6 g/dl. White blood cell count (WBC), platelet (PLT) levels and coagulation profile were normal.

Vascular injury was suspected and an urgent angiography was arranged. Unfortunately on the way to the radiology department, the wound started bleeding massively. The patient's systolic blood pressure dropped to 40mmHg and heart rate rose to 132 pulses/minute. Peripheral pulses could not be detected and she was initially confused and finally unresponsive. Ringer's lactate and units of whole blood were immediately transfused, and the patient, being in obvious hemorrhagic shock, was taken urgently to the operating room.

**Figure 3:**
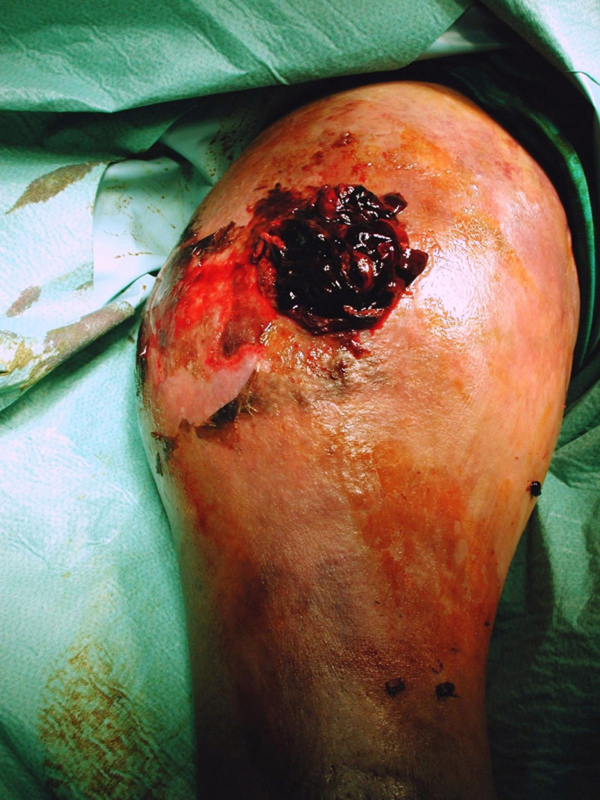
**A large amount of thrombus filling the false aneurysm**.

The fracture site was exposed via a lateral incision on her right arm. The entire area from the elbow almost to the shoulder was full of blood clots (Figure 3) and the cavity was lined with grayish, glistening membrane. The actual aneurysmatic cyst of the brachial artery was revealed to be 4cm × 5cm in size at the level of the fracture. The adjacent muscles were found to be necrotic and had mainly been replaced by fibro fatty tissues due to severe ischemia caused by the prolonged pressure. The opening on the arterial wall was fissure-like, 1cm long with smooth edges. Taking into consideration that the patient was very old, was in a critical condition, and that the fracture had been grossly displaced for 2 months and obviously contaminated due to the presence for 3 weeks of a 2 by 3cm ulcer (more or less septic pseudarthrosis) as well as the poor quality of the adjacent soft tissues, including necrotic muscles, it was decided to amputate the arm above the elbow. This was also the opinion of the senior vascular surgeon at our hospital. The patient received 7 units of whole blood perioperatively and the final Hb was 7g/dl.

The day after the operation, the patient's condition had greatly improved, she was alert, hemodynamically stable, ambulatory and afebrile. The wound healed uneventfully and at follow-up examinations, she was quite happy even with the amputated arm and she refused the offer of a prosthesis.

## Discussion

Fractures of long bones rarely have vascular complications. The axillary and brachial artery may be injured after a humeral fracture, or after an anterior shoulder dislocation [4,5]. To the best of our knowledge, there is no report in the literature of a false aneurysm of the brachial artery, particularly as a delayed complication of a periprosthetic humeral fracture.

An aneurysm is formed over weeks or even months [2]. Classically, a false aneurysm appears as a pulsatile swelling at the fracture site. Increased intracompartmental pressure and associated venous edema may cause skin necrosis and subsequent ulceration [4]. Meanwhile, peripheral pulses can be preserved by the collateral blood supply [3] and even Allen's test is negative: findings that can delay the diagnosis. However, if large pieces of thrombus enter the pseudoaneurysm, thrill and pulsatility will be undetectable clinically [1], as happened in our patient.

Due to their clinical appearance, peripheral artery aneurysms of the extremities can be easily misdiagnosed as hematomas or even as soft tissue tumors. In addition, pressure and hyperemia can result in resorption of adjacent bone. A biopsy in such cases may be hazardous [7,8]. The history of trauma (recent or even previous) in conjunction with progressive soft tissue swelling should alert the clinician to a potential vascular injury as a differential diagnosis.

Plain radiographs can show enormous displacement at the fracture site and the diagnosis is usually established by angiography. Arterial Doppler ultrasonography can be used in the diagnostic procedure as it is a noninvasive, low-cost and usually available imaging method [7,9]. Magnetic resonance imaging (MRI) can also be used; an aneurysm appears on both T1- and T2-weighted images and the use of intravenous gadolinium does not enhance the signal [7].

However, the gold standard of diagnostic tools is classic angiography and especially selective arteriography [9]. Selective catheterization of the injured artery allows not only the detection of the aneurysm, but also the pre-operative embolization if there is a feeding artery [7].

Unfortunately, we were not able to proceed with any of these investigations because the patient started bleeding massively on the way to the radiology department. Diagnosis was confirmed intra-operatively. The removal of the U-slab had left the periprosthetic fracture unstable and obviously the size of the false aneurysm had greatly increased. Diagnosis was not established earlier because the intact neurological function in the extremity and the presence of normal perfusion of the hand along with a palpable radial and ulnar pulse created a false sense of security regarding arterial competence. However, the severe displacement at the fracture site, revealed by radiography, should have raised the suspicion of the presence of the false aneurysm from the beginning.

## Conclusions

In cases of blunt trauma, gunshot injury, or fracture of the humerus with increasing displacement at the fracture site, excessive local swelling and anemia, and despite the presence of normal perfusion of the hand and palpable radial and ulnar pulses, the surgeon must rule out the possibility of the presence of a false aneurysm to avoid the extremely unpleasant situation of having to perform an amputation as a salvage procedure.

## Consent

Written informed consent was obtained from the patient for publication of this case report and any accompanying images. A copy of the written consent is available for review by the Editor-in-Chief of this journal.

## Competing interests

The authors declare that they have no competing interests.

## Authors' contributions

EP operated on the patient and supervised writing of the case report. EA wrote this article contributed in the concept and methodology of the essay and took part in the operation. CM took part in the operation, contributed in data analysis, interpretation of material and reference gathering and also assisted in formulation of the text, while GK searched the relevant bibliography and followed up the patient. JG supervised the writing of the discussion. IT provided useful information on aneurysms of peripheral arteries and took part in the operation. All authors read and approved the final manuscript.
